# Two-Year Outcomes of the Nellix EndoVascular Aneurysm Sealing System for Treatment of Abdominal Aortic Aneurysms

**DOI:** 10.1177/1526602818766864

**Published:** 2018-03-29

**Authors:** Aleksandra C. Zoethout, Johannes T. Boersen, Jan M. M. Heyligers, Jean-Paul P. M. de Vries, Clark J. A. M. Zeebregts, Michel M. P. J. Reijnen

**Affiliations:** 1 Department of Vascular Surgery, Rijnstate Hospital, Arnhem, the Netherlands; 2 Department of Surgery, Division of Vascular Surgery, University Medical Center Groningen, University of Groningen, the Netherlands; 3 Department of Vascular Surgery, St Antonius Hospital, Nieuwegein, the Netherlands; 4 Department of Vascular Surgery, Elisabeth-TweeSteden Hospital, Tilburg, the Netherlands

**Keywords:** abdominal aortic aneurysm, endovascular aneurysm repair, endovascular aneurysm sealing, instructions for use

## Abstract

**Purpose:** To analyze the 2-year outcomes of endovascular aneurysm sealing (EVAS) according to 2 versions of the instructions for use (IFU). **Methods:** A retrospective study was conducted involving 355 consecutive patients treated with the first-generation EVAS device from April 2013 to December 31, 2015, at 3 high-volume centers. Out of 355 patients treated with EVAS, 264 were elective asymptomatic infrarenal EVAS procedures suitable for analysis. In this cohort, 168 (63.3%) patients were treated within the IFU 2013 criteria; of these 48 (18.2%) were in compliance with the revised IFU 2016 version. **Results:** Overall technical success was 98.2% (165/168) in the IFU 2013 group and 97.9% (47/48) in the IFU 2016 subgroup (p=0.428). The 2-year freedom from reintervention estimates were 89.7% (IFU 2013) and 95.7% (IFU 2016), with significantly more reinterventions in the first 45 cases (p=0.005). The stenosis/occlusion estimates were 6.5% (IFU 2013) and 4.2% (IFU 2016; p=0.705). Nine (5.4%) endoleaks (8 type Ia and 1 type Ib) were observed within the IFU 2013 cohort; 3 (2.1%) were in the IFU 2016 subgroup (p=0.583). Migration ≥10 mm or ≥5 mm requiring intervention was reported in 12 (7.1%) patients in the IFU 2013 cohort but none within the IFU 2016 subgroup. Ten (6.0%) patients demonstrated aneurysm growth in the IFU 2013 cohort, of which 2 (4.2%) were in the IFU 2016 subgroup. Overall survival and freedom from aneurysm-related death estimates at 2 years were 90.9% and 97.6% in the IFU 2013 cohort (IFU 2016: 95.5% and 100.0%). The prevalence of complications seemed lower within IFU 2016 without significant differences. **Conclusion:** This study shows acceptable 2-year results of EVAS used within the IFU, without significant differences between the 2 IFU versions, though longer follow-up is indicated. The refined IFU significantly reduced the applicability of the technique.

## Introduction

Endovascular aneurysm repair (EVAR) has become the most frequently used treatment modality for infrarenal abdominal aortic aneurysms (AAAs) because of reduced early morbidity and mortality, but the benefit of EVAR over open repair declines after 2 years owing to endoleak, graft displacement, limb occlusions, stent fractures, and secondary ruptures.^1–6^ In addition to a linear pullout force, lateral movement of the endograft leads to endograft instability and late adverse events, but none of the current EVAR devices are designed to oppose these forces.^7^

The Nellix EndoVascular Aneurysm Sealing System (Endologix, Inc, Irvine, CA, USA) was developed in an attempt to reduce complications, particularly endoleaks and subsequent reinterventions.^8^ The EVAS device consists of dual balloon-expandable stent-grafts surrounded by endobags that are filled in situ with polymer to achieve seal and anatomical fixation.^9,10^ Since its launch in 2013, the Nellix device has shown a high aneurysm exclusion rate and a low frequency of complications,^8,11–17^ but concerns exist as regards migration and endoleak in the long term.

Although commercially available EVAR stent-grafts come with device-specific instructions for use (IFU), these instructions are oftentimes set aside due to clinical necessity and possible misjudgment of device capacities. EVAR studies have shown that the more the IFU are violated, the more the repair is prone to failure.^18,19^ This emphasizes the importance of selecting patients with proper aneurysm morphology as a key determinant for successful exclusion of the aneurysm.

Current early results on EVAS are based on study populations consisting of patients treated within and outside the Nellix IFU, which could have affected results. In the beginning, EVAS appeared to be applicable to a wider range of aneurysm morphologies than other devices then in use, according to the original IFU (Table 1).^20^ In the EVAS FORWARD Global IDE Trial,^16^ 1-year safety and effectiveness results demonstrated low morbidity and mortality and high procedural and treatment success. However, 2-year imaging revealed a signal of migration, leading to a dedicated root cause analysis traced to lateral bending^21,22^ of the stents into surrounding soft thrombus. The ability of the stents themselves to resist bending forces is low, requiring polymer support. To address complications (especially migration but also endoleak and AAA sac growth) and improve outcomes, the IFU were refined in 2016 based on unpublished 2-year results of the EVAS IDE trial. The changes include a reduced maximum infrarenal neck diameter and reduced degree of neck diameter change from ≤20% to ≤10% (Table 1). Also, an aortic lumen ratio was added to the IFU, comparing the maximum diameter of the aortic aneurysm to the maximum aortic blood lumen diameter. This limits the proportion of thrombus allowed in the aneurysm in order to provide better seal and prevent migration. Finally, distal seal criteria were made more stringent; the maximum iliac artery diameter was limited to 20 mm and a distal seal zone length was added. The aim of the present study was to retrospectively analyze the 2-year outcomes of the EVAS device based on the 2013 and 2016 versions of the IFU.

**Table 1.** Instructions for Use (IFU) for the Nellix EndoVascular Aneurysm Sealing System.

IFU 2013	Refined IFU 2016
Infrarenal neck length ≥10 mm	No change^a^
Proximal neck diameter change ≤20%	≤10%
Proximal neck diameter 18 to 32 mm	18 to 28 mm
Infrarenal neck angle ≥60°	No change
Aneurysm blood lumen diameter ≤60 mm	No change
	Ratio of maximum aortic aneurysm diameter to maximum aortic blood lumen diameter <1.4
Iliac artery luminal diameter 9 to 35 mm	9 to 20 mm
	Distal iliac artery seal zone length ≥10 mm with maximal 25 mm diameter (inner to inner wall)
Femoral access ≥7 mm	No change

a Definition for location of distal end of neck changed from 10% increase from the diameter of the lowest renal artery in 2013 to 20% in 2016.

## Methods

### Study Design

**Figure 1.**
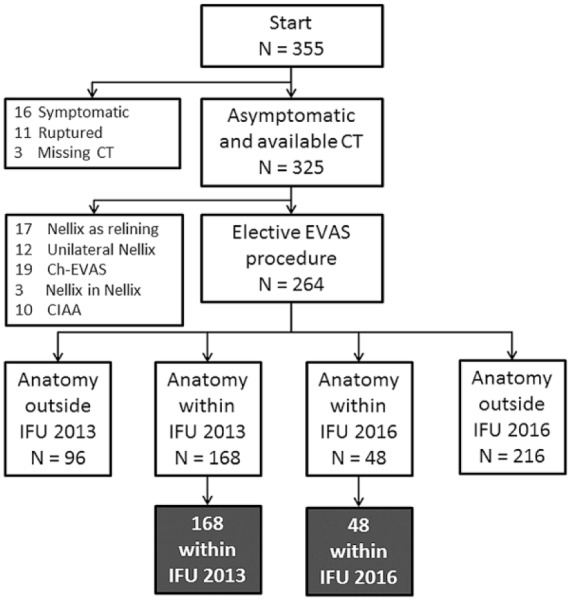
Flowchart of patient exclusion and assignment to within or outside the original (2013) and revised (2016) instructions for use (IFU). Note that the IFU 2016 patients are a subgroup of the IFU 2013 cohort, and several patients were excluded based on >1 anatomical criterion (see Table 3). Ch-EVAS, chimney endovascular aneurysm sealing; CT, computed tomography; EVAS, endovascular aneurysm sealing.

A retrospective cohort study was conducted of all patients treated with the first-generation Nellix (3SQ+) from April 2013 to December 31, 2015, in 3 high-volume centers in the Netherlands, each performing >100 EVAS procedures within that time frame. Patients were eligible for this analysis if they had an asymptomatic infrarenal AAA treated with a standard 2-component Nellix device in an elective procedure. Of the 355 patients treated in the observation period, 27 were excluded because they were treated for a symptomatic (n=16) or ruptured (n=11) AAA (Figure 1). Three more patients were excluded because of inaccurate sizing on magnetic resonance angiography in 3Mensio or because of missing preoperative computed tomography (CT) scans (patients referred from other centers). From the remaining 325 patients, 61 were excluded because they were not treated with a regular EVAS procedure, including EVAS as relining after previous EVAR (n=17), unilateral EVAS (n=12), chimney EVAS (ch-EVAS; n=19), Nellix-in-Nellix application (n=3), or EVAS for an isolated CIA aneurysm (n=10). This left 264 asymptomatic patients electively treated for an infrarenal AAA in a standard EVAS procedure. Ninety patients included in the analysis had been part of the EVAS FORWARD Global registry^15^ or the EVAS FORWARD IDE trial.^16^

The study was conducted according to the principles of the Declaration of Helsinki and in accord with the applicable national guidelines, regulations, and acts. Personal data were anonymized and handled in compliance with the Dutch Personal Data Protection Act. The Dutch central ethics board waived the need for informed consent for a retrospective review.

### Patient Selection and IFU Assignment

Hospital records of the 264 patients were reviewed and relevant data retrieved; comorbidities were scored according to the Society for Vascular Surgery (SVS) comorbidity grading scale.^23^ Pre- and postoperative computed tomography angiography (CTA) scans were assessed by an independent observer (J.T.B.) with the use of 3-dimensional (3D) vascular planning software (3Mensio Vascular; Pie Medical Imaging, Bilthoven, the Netherlands) to determine compliance with the IFU 2013 and IFU 2016. Diameter measurements were based on an outer to outer vessel diameter perpendicular to the center lumen line (CLL) or, if specified, the flow lumen diameter (excluding intraluminal thrombus). Aortic neck diameters and common iliac artery (CIA) flow lumen diameters were calculated as the mean of the anteroposterior and lateral diameters. The maximum aneurysm and CIA artery diameters and maximum aortic flow lumen diameter included a single measurement of the largest diameter perpendicular to the CLL.

The maximum aneurysm and maximum aortic flow lumen diameters were used to calculate the diameter ratio for the refined IFU 2016. Vascular access was adequate when the external iliac artery (EIA) diameter was >7 mm. The infrarenal neck length was measured by the centerline distance from the baseline (ie, lowest renal artery) to the nearest plane that shows a 20% (IFU 2013) or 10% (IFU 2016) increase in aortic diameter over the diameter at baseline. Suprarenal and infrarenal angulations were measured in accord with a previously reported standard.^24^ A distal seal zone was determined by any vessel segment in the CIA or EIA with an outer to outer diameter in a range of 9 to 20 mm and a minimum length of 10 mm within a length of 170 mm from the baseline, defined by the sealing length (maximum EVAS stent length 180 mm for the first-generation Nellix EVAS system). Preoperative and postoperative seal lengths were assessed in 3D CT analysis to determine the placement of the endosystems. Of the 264 patients reviewed, 168 (63.6%) had anatomical characteristics within the IFU 2013, while 48 (18.2%) of these patients were within the refined IFU 2016. The baseline and anatomical characteristics of these study groups are shown in Table 2 and a breakdown of the compliance with both IFU versions in Table 3.

**Table 2.** Baseline and Anatomical Characteristics.^a^

Variable	Total Cohort (n=264)	IFU 2013 (n=168)	IFU 2016 Subgroup (n=48)	p^b^
Demographics and comorbidities				
Age, y	75 (68, 79)	74 (68, 79)	75 (68.2, 79)	0.702
Men	310 (87.3)	155 (92.3)	40 (83.3)	0.064
ASA class				0.766
II	199 (56.1)	107 (63.7)	29 (60.4)	
≥III	146 (41.1)	60 (35.7)	18 (37.5)	
Missing	10 (2.8)	1 (0.6)	1 (2.1)	
Hypertension	242 (68.2)	112 (66.7)	33 (68.8)	0.786
Hyperlipidemia	267 (75.2)	126 (75.0)	38 (79.2)	0.552
Smoking (in past 10 years)	159 (44.8)	78 (46.4)	20 (41.7)	0.559
Cardiac disease	151 (42.5)	72 (42.9)	27 (56.3)	0.117
Pulmonary disease	96 (27.0)	47 (28.0)	13 (27.1)	0.903
Renal disease^c^	91 (25.6)	34 (20.2)	8 (16.7)	0.581
Diabetes mellitus	51 (14.4)	27 (16.1)	11 (22.9)	0.272
Anatomical characteristics				
Infrarenal neck diameter, mm	23.4 (24.4, 26.0)	23.3 (21.8, 25.5)	22.3 (20.9, 24.3)	0.031
Infrarenal neck angle, deg	23.0 (12.5, 35.6)	21.9 (13.1, 35.0)	25.2 (15.2, 38.2)	0.234
Infrarenal neck length at 10% diameter increase, mm	16 (9.8, 30.0)	18.0 (12.0, 31.0)	20.0 (15.0, 30.8)	0.548
AAA lumen diameter, mm	40.9 (36.2, 48.3)	42.3 (37.9, 48.1)	46.6 (41.3, 50.8)	0.003
AAA outer diameter, mm	57.7 (54.0, 62.7)	57.9 (54.3, 61.7)	56.4 (53.0, 61.2)	0.141
Ratio AAA outer diameter to AAA lumen diameter	1.37 (1.19, 1.60)	1.35 (1.19, 1.57)	1.27 (1.15, 1.32)	0.000
Infrarenal lumen volume, mL	78.3 (59.0, 107.1)	80.3 (64.1, 107.3)	93.6 (73.7, 110.8)	0.128
Right CIA lumen diameter, mm	10.0 (9.0, 11.8)	10.5 (9.3, 12.0)	10.0 (9.2, 11.0)	0.054
Right CIA outer diameter, mm	17.4 (14.3, 21.4)	18.0 (15.0, 21.4)	16.5 (14.1, 18.2)	0.002
Left CIA lumen diameter, mm	10.0 (9.0, 11.8)	10.5 (9.3, 12.0)	10.2 (9.1, 11.0)	0.133
Left CIA outer diameter, mm	17.1 (14.0, 20.7)	17.2 (14.8, 20.4)	16.0 (14.1, 18.1)	0.019

Abbreviations: AAA, abdominal aortic aneurysm; ASA, American Society of Anesthesiologists; CIA, common iliac artery; IFU, instructions for use.

a Continuous data are presented as the median (interquartile range Q1, Q3); categorical data are given as the counts (percentage).

b Comparison between the IFUs.

c Creatinine level ≥2.4 mg/dL.

**Table 3.** Compliance With the Nellix Instructions for Use in 2013 and 2016 for the 264 Electively Treated Patients.^a,b^

Instructions for use 2013	
Infrarenal neck	222 (84.1)
Length ≥10 mm and ≤20% diameter change	233 (88.3)
Diameter ≥18 mm	254 (96.2)
Diameter ≤32 mm	244 (92.4)
Missing data	4 (1.5)
Infrarenal neck angle ≤60°	258 (97.7)
Aneurysm blood lumen diameter ≥60 mm	254 (96.2)
CIA lumen diameter 9 to 35 mm	207 (78.4)
CIA diameter ≥9 mm	206 (78.0)
CIA diameter ≤35 mm	264 (100.0)
Missing data	5 (1.9)
Access diameter >7 mm	236 (89.4)
Instructions for use 2016	
Infrarenal neck	183 (69.3)
Length ≥10 mm and ≤10% diameter change	204 (77.3)
Diameter ≥18 mm	254 (96.2)
Diameter ≤28 mm	207 (78.4)
Missing data	3 (1.1)
Infrarenal neck angle ≤60°	258 (97.7)
Aneurysm blood lumen diameter ≤60 mm	254 (96.2)
Ratio of maximum aneurysm diameter to maximum blood lumen diameter <1.4	149 (56.4)
CIA lumen diameter 9 to 35 mm	207 (78.4)
CIA diameter ≥9 mm	206 (78.0)
CIA diameter ≤35 mm	264 (100.0)
Missing data	5 (1.9)
Distal iliac artery seal (length ≥10 mm and outer diameter 9 to 20 mm)	167 (63.3)
Access diameter >7 mm	236 (89.4)

Abbreviation: CIA, common iliac artery.

a Data are presented as the count (percentage).

b Note that some of the patients were out of compliance with >1 criterion.

### EVAS Procedure and Follow-up

The EVAS procedure has been detailed previously.^9^ After antibiotic prophylaxis, bilateral femoral accesses were obtained either percutaneously or by surgical cutdown and heparin was administered. The Nellix stent-grafts were inserted bilaterally over stiff wires and preferably positioned just distal to the renal arteries. Once the sheaths were retracted, a vacuum was applied to test the integrity of the endobags. Subsequently, the balloons in the stents were inflated simultaneously to 7 atm. The endobags were prefilled with saline to verify the calculated polymer quantity and to assess the absence of endoleaks with the intended endobag pressure of 180 mm Hg. The endobags were deflated, and a polyethylene glycol–based polymer was instilled into the endobags with a filling pressure aimed at 180 mm Hg while the position was maintained. If required a secondary fill was performed to correct insufficient sac filling.^9^

Various modifications in the treatment protocol were implemented during the observation period: (1) the intended endobag pressure was higher than 180 mm Hg (220–250 mm Hg), (2) after endobag filling, the Nellix stents were postdilated with 10-mm balloons, and (3) the Nellix balloons were left inflated during the entire procedure or reinflated during the curing process. These modifications were done to increase stability and to avoid pillowing of the endobags into the interior of the stent frames.

Follow-up involved outpatient clinic visits at 1 to 3 months, 12 months, and annually thereafter unless events required closer examination. A window of 2 months was added to the 2-year follow-up, extending the total follow-up to 26 months to ensure no data were missed due to scheduling. Follow-up consisted of clinical examination, CTA and/or duplex ultrasound imaging, and abdominal radiography. In 32 patients (10 within IFU 2016), only the first postoperative CT scan was available.

### Endpoints and Definitions

The main study endpoint was reintervention for any type of endoleak, device stenosis or occlusion, device migration, AAA growth, or device defect within 2 years of the initial procedure. The secondary outcomes were technical success, survival, aneurysm-related death, endoleak, limb stenosis/occlusion, migration, and AAA growth.

The outcome measures were defined according to the SVS reporting standards.^25^ Technical success was defined as successful introduction and deployment of the device without conversion, death, type I or III endoleak, or graft limb occlusion within 24 hours after the procedure. The term “assisted technical success” was applied to cases in which unplanned endovascular or surgical procedures were required to successfully exclude the aneurysm. Primary patency referred to freedom from stenosis or occlusion without the need for other surgical or endovascular procedures; secondary patency was obtained with the use of a surgical or endovascular procedure after occlusion occurred.^25^ Based on the reporting standards,^25^ migration was defined as ≥10-mm caudal displacement of one or both EVAS stent-grafts compared to the first postoperative CT or ≥5-mm migration that required a reintervention. In addition to migration, the current analysis recorded caudal movement of 5 to 10 mm.

Endoleaks were classified according to the reporting standards,^25^ but proximal type I endoleaks were subclassified using a new EVAS-specific system^26^: type Is1 refers to the appearance of contrast between the endobag and the wall of the proximal neck but not in continuation with the aneurysm sac, type Is2 is visible contrast in between the endobag and aneurysm wall or thrombus, and type Is3 shows contrast or fresh thrombus in between the endobags inside the sac.

Aneurysm growth was assessed by >5-mm increase in maximum aneurysm diameter between the last available follow-up scan within 2 years and the first postoperative CT. If patients were followed by duplex ultrasound only, the diameter was compared to the diameter of the first postoperative ultrasound. Migration and caudal movement were reported based only on the 3D CT analysis, while endoleaks and aneurysm growth were reported based on all follow-up imaging. All endoleaks diagnosed on duplex were confirmed on CT. If an endoleak was witnessed on ultrasound but not on CT, the CT data were regarded as superior. Aneurysm-related mortality included death due to aneurysm rupture or the consequences of a primary or secondary procedure or surgical conversion.

### Statistical Analysis

Continuous variables were presented as the mean ± standard deviation or as median and interquartile range (IQR: Q1, Q3) depending on the results of normalcy testing. Categorical variables were presented as frequencies and percentages. Proportions and nominal variables were compared using the chi-square test or the Fisher exact test in the case of a small sample size. Continuous variables were compared by means of the independent *t* test or the Mann-Whitney *U* test for nonparametric data.

Kaplan-Meier analysis was employed to estimate rates for survival, freedom from reintervention, primary and secondary patency, freedom from migration, freedom from AAA growth, freedom from any endoleak (type Ia/b, II, and III), and freedom from type Ia endoleak (Is2 and Is3). Curves were compared with the log-rank test. Datasets were truncated when the standard error exceeded 10%. The threshold of statistical significance was p<0.05. All statistical analyses were performed using IBM SPSS Statistics (version 24.0; IBM Corporation, Armonk, NY, USA).

Since this study included all patients treated within the IFU since the introduction of the Nellix device, a subanalysis was performed to determine whether the results were influenced by the learning curve. Based on previous research showing that optimal EVAR results are achieved after 10 to 20 cases,^27^ the first 15 cases (based on the date of the EVAS procedure) within the IFU 2013 in each center were regarded as the learning phase and compared with the remainder of cases within the IFU 2013 group. Another IFU 2013 subgroup analysis was performed comparing the infrarenal neck length in those patients who experienced an endoleak, aneurysm growth, migration, and/or caudal movement of 5 to 10 mm to the patients who had no complication.

## Results

There were no statistically significant differences in baseline characteristics between the IFU 2013 patients and the IFU 2016 subgroup (Table 2). However, because of the change in anatomical requirements in 2016, significant differences in anatomical variables were observed between the IFU 2013 and IFU 2016 versions. The median infrarenal neck diameter was 23.3 mm (IQR 21.8, 25.5) within the IFU 2013 and 22.3 mm (IQR 20.9, 24.3) within the IFU 2016 (p=0.031). Also, the AAA lumen diameter (p=0.003) and the ratio of the AAA outer lumen diameter to AAA lumen diameter (p<0.001) were significantly different, as were the CIA outer diameters on both sides (right p=0.002 and left p=0.019). No other significant differences between the IFUs were observed.

The majority of patients were operated upon under general anesthesia (p=0.583) with surgical cutdown of the CIA (p=0.782). Distal extensions were used in 15 (8.9%) patients in the IFU 2013 cohort, of which 7 (4.2%) were planned preoperatively; there was only 1 (2.1%) distal extension within the IFU 2016 (p=0.272) that was not planned preoperatively.

Overall technical success was 98.2% (165/168) in the IFU 2013 group and 97.9% (47/48) in the IFU 2016 subgroup (p=0.428). Of the 9 assisted technical success cases in the larger cohort, 8 were due to an unplanned distal extension; in the other case, an endobag had lost integrity after the prefill and the other endobag was used to fill the aneurysm sac. The technical failures in both groups were due to postoperative proximal type Ia endoleaks. One type II endoleak was observed at completion angiography. All immediate postoperative endoleaks were not observed during further follow-up. Access-related complications are shown in Table 4. One patient had renal function deterioration not requiring dialysis after protrusion of the endobag over a renal artery. No other adverse events were observed during the hospital stay.

**Table 4.** Perioperative Data.^a^

Variable	IFU 2013 (n=168)	IFU 2016 Subgroup (n=48)	p^b^
Procedure			
Anesthesia (general)	143 (85.1)	36 (75.0)	0.583
CIA cutdown	164 (97.6)	46 (95.8)	0.782
Blood loss, mL	130 (100, 300)	150 (63, 300)	0.939
Duration, min	90 (70, 108)	90 (74, 106)	0.915
Hospital stay, d	3 (3, 4)	3 (3, 4)	0.323
Technical success	165 (98.2)	47 (97.9)	0.428
Primary	156 (92.9)	46 (95.8)	
Assisted	9 (5.4)	1 (2.1)	
Complications			
Type Ia endoleaks	3 (1.8)	1 (2.1)	
Renal	1 (0.6)	1 (2.1)	
Access-related			
Groin hematoma	11 (6.5)	4 (8.3)	0.437
Groin swelling	8 (4.8)	4 (8.3)	0.264
Neuralgia	8 (4.8)	1 (2.1)	0.367
Wound leak	2 (1.2)	0	0.604
Wound infection	2 (1.2)	1 (2.1)	0.531

Abbreviations: CIA, common iliac artery; IFU, instructions for use.

a Continuous data are presented as the median (interquartile range Q1, Q3); categorical data are given as the counts (percentage).

b Comparison between the IFUs.

### Outcomes at 30 Days and in Follow-up

In both IFU groups the median follow-up was 23 months (IFU 2013 IQR: 12, 26; IFU 2016 IQR 12, 29; p=0.495). At 1 year, 119 of the 168 IFU 2013 patients and 26 of the 48 IFU 2016 patients were present for follow-up. Seventy of the IFU 2013 patients and 23 of the IFU 2016 patients had ≥2 years of follow-up. Within the entire cohort the median follow-up was 18 months (IQR 11, 24). The available imaging modality at each follow-up period for the IFU 2013 cohort is reported in Table 5.

**Table 5.** Imaging Modality per Follow-up Period for the Patients Within the 2013 Nellix Instructions for Use.^a^

Type of Imaging	0 to 6 Months (n=168)	6 to 12 Months (n=157)	12 to 18 Months (n=138)	18 to 24 Months (n=107)
CTA	57 (33.9)	34 (21.7)	35 (25.4)	31 (29.0)
CTA and duplex	40 (23.8)	14 (8.9)	10 (7.2)	9 (8.4)
CTA and radiography	10 (6.0)	3 (1.9)	4 (2.9)	2 (1.9)
CTA, duplex, and radiography	42 (25.0)	19 (12.1)	11 (8.0)	9 (8.4)
Duplex	13 (7.7)	31 (19.7)	19 (13.8)	18 (16.8)
Duplex and radiography	0 (0)	5 (3.2)	2 (1.5)	1 (0.9)
Missing data	6 (3.6)	51 (32.5)	57 (41.3)	37 (34.6)

a Data are presented as the count (percentage).

Abbreviations: CTA, computed tomography angiography.

#### Reinterventions

During the 2-year follow-up, a total of 19 (11.3%) patients required a reintervention in the IFU 2013 group vs 4 (8.3%) in the IFU 2016 subgroup (p=0.555; Table 6). Three (1.8%) IFU 2013 patients had a reintervention within 30 days for occlusions or stenoses, including a femoral-femoral bypass, thrombectomy and relining of the endoprosthesis, and thrombectomy of the Nellix system in combination with common femoral artery endarterectomy. One (2.1%) patient in the revised IFU group required a femoral-femoral bypass.

**Table 6.** Outcomes for Different Follow-up Periods and Both IFU Cohorts.^a^

Outcome	<30 Days		30 Days to 1 Year		1 to 2 Years	
	IFU 2013 (n=168)	IFU 2016 (n=48)	IFU 2013 (n=119)	IFU 2016 (n=26)	IFU 2013 (n=70)	IFU 2016 (n=23)
Reinterventions	3 (1.8)	1 (2.1)	6 (5.0)	1 (3.8)	10 (14.3)	2 (8.7)
Conversions	—	—	1 (0.8)	—	5 (7.1)	1 (4.3)
Occlusion	5 (3.0)	2 (4.2)	1 (0.8)	—	2 (2.9)	—
Stenosis	1 (0.6)	—	2 (1.7)	—	—	—
Endoleak^b^						
Type Is2	3 (1.8)^c^	1 (2.1)^c^	2 (1.7)	1 (3.8)	3 (4.3)	—
Type Is3	—	—	1 (0.8)	—	2 (2.9)	2 (8.7)
Type Ib	—	—	1 (0.8)	—	—	—
Type II	1 (0.6)^c^	—	—	—	—	—
Migration	—	—	2 (1.7)	—	10 (14.3)	—
Caudal movement	—	—	10 (8.4)	4 (15.4)	15 (21.4)	
Sac growth	—	—	2 (1.7)	—	6 (8.6)	2 (8.7)

Abbreviations: IFU, instructions for use.

a Data are presented as the count (percentage).

b According to van den Ham et al.^24^

c These intraoperative leaks were not visible on postoperative imaging.

Of the 7 reinterventions performed between 30 days and 1 year, 1 (2.1%) was in the IFU 2016 group (thrombectomy with relining) and 6 (3.6%) were in the IFU 2013 group (1 thrombectomy with relining, 2 thrombectomies with iliac extension/stenting, 1 thrombolysis with iliac stenting, 1 relining of both EVAS stents for iliac stenosis, and 1 conversion to open repair due to an aortoenteric fistula 3 months after the EVAS procedure).

At 2-year follow-up, 10 (6.0%) additional reinterventions were performed within the IFU 2013 cohort, including relining for a stenosis, femoral-femoral bypass for occlusion, embolization for a type Ia endoleak, and 2 Nellix-in-Nellix proximal extensions for a secondary rupture and a migration, respectively. The other 5 IFU 2013 patients underwent conversion to open repair owing to type Ia endoleak with migration at 24 months, sac expansion at 15 months, periaortitis at 22 months, and 2 for type Ia endoleak at 13 and 24 months. Two of the reinterventions for type Ia endoleak (embolization and conversion) involved patients in the IFU 2016 subgroup. In total, 6 conversions to open repair were performed within the 2-year period after the primary Nellix procedure.

**Figure 2.**
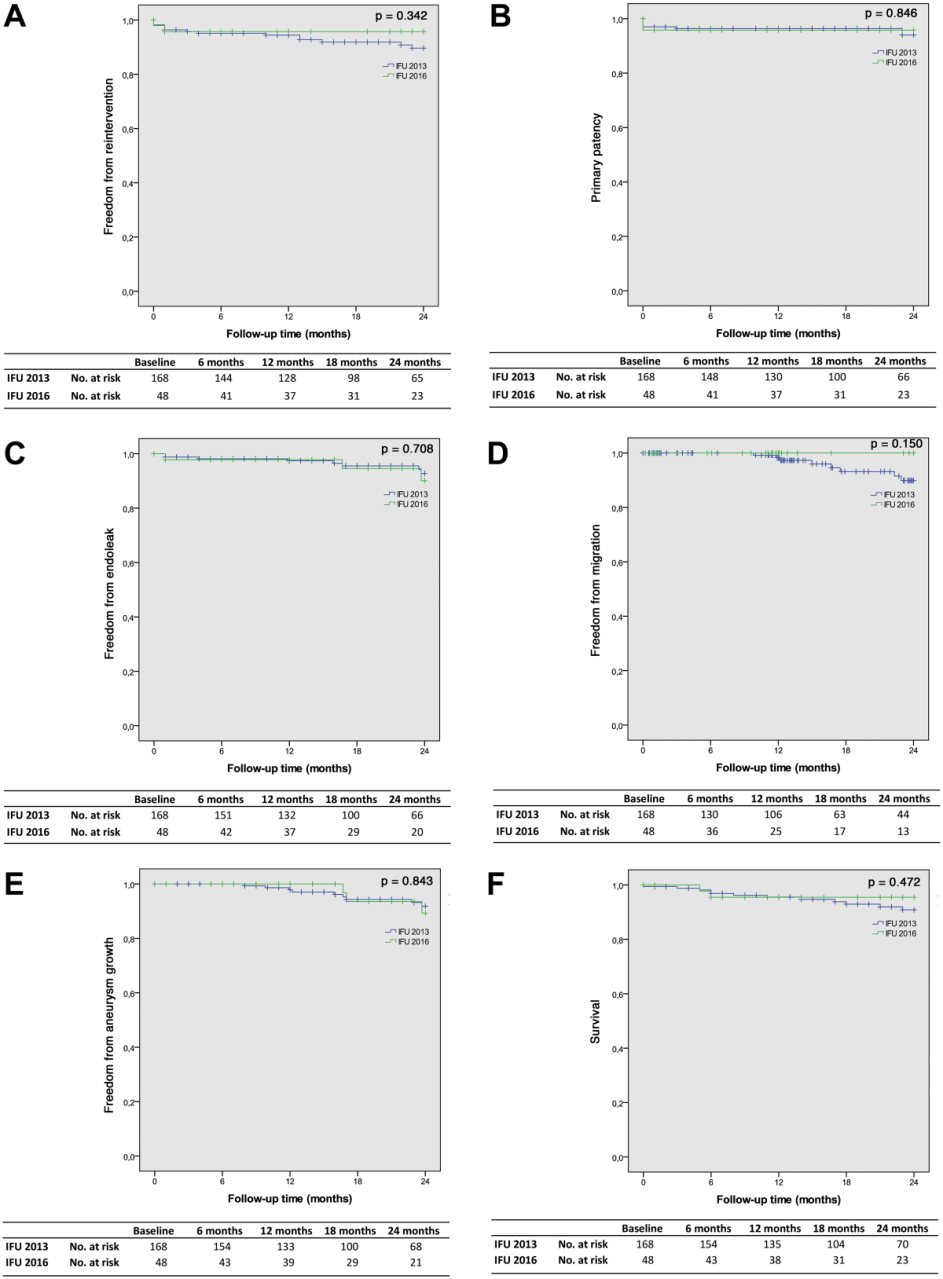
Kaplan-Meier analyses of (A) freedom from reintervention, (B) primary patency, (C) freedom from any endoleak, (D) freedom from migration, (E) freedom from aneurysm growth, and (F) overall survival. The standard error did not exceed 10% at any time point in all curves.

The freedom from reintervention estimates were 94.4% (95% CI 90.9% to 97.9%) and 89.7% (95% CI 84.4% to 95.0%) at 1 and 2 years for the 2013 IFU and 95.7% (95% CI 90.0% to 100%) for both time points in the 2016 IFU subgroup, respectively (p=0.342; Figure 2A).

#### Stenosis/occlusion

There were 11 (6.5%) patients with a symptomatic stenosis or occlusion (Table 6) during the 2-year follow-up in the IFU 2013 group and 2 (4.2%) in the IFU 2016 subgroup (p=0.705). Five (3.0%) occlusions (2 in IFU 2016 patients) and 1 (0.6%) symptomatic stenosis were observed at ≤30 days. Two of these patients presented with a reocclusion at 1 year and both were treated with relining using a self-expanding covered stent. At 1 year, there were 2 new-onset symptomatic stenoses and 1 new-onset occlusion; all underwent a reintervention. At 2 years there were 2 new-onset occlusions reported. One patient was planned for a reintervention, but this was not yet performed within 26 months. The other patient with an occlusion was considered high risk for reintervention and was treated conservatively with physical therapy. Overall, all but 2 patients with a symptomatic stenosis or occlusion received a reintervention.

The primary patency estimates for the IFU 2013 cohort were 96.4% (95% CI 93.7% to 99.1%) and 94.0% (95% CI 89.7% to 98.3%) at 1 and 2 years, respectively (Figure 2B); for the IFU 2016 subgroup both estimates were 95.8% (95% CI 90.1% to 100%; p=0.846). The secondary patency estimates were 100% at 1 year and 97.6% (95% CI 94.5% to 100%) at 2 years for the IFU 2013 patients and 100% at both time points for the IFU 2016 subgroup (p=0.432).

#### Endoleak

Overall, 9 (5.4%) endoleaks (Table 6) were observed in the IFU 2013 cohort during the 2-year follow-up, of which 3 (2.1%) were in the IFU 2016 subgroup (p=0.583). Four endoleaks were found between 30 days and 1 year within the IFU 2013 cohort and another 5 between 1 and 2 years. One endoleak was seen between 30 days and 1 year and 2 endoleaks were seen between 1 and 2 years in the IFU 2016 subgroup. At 2 years, 5 of 9 endoleaks had received a reintervention as detailed previously. The freedom from any endoleak estimates (Figure 2C) at 1 and 2 years were 97.4% (95% CI 95.2% to 99.9%) and 92.7% (95% CI 87.6% to 97.8%), respectively, for the IFU 2013 cohort and 97.8% (95% CI 93.5% to 100%) and 90.1% (95% CI 78.9% to 100%) for the IFU 2016 subgroup (p=0.708). Freedom from type Ia endoleak estimates at the same time points were 98.0% (95% CI 95.8% to 100%) and 93.3% (95% CI 88.2% to 98.4%) for the patients in the IFU 2013 group and 97.8% (95% CI 93.5% to 100%) and 90.1% (95% CI 78.9% to 100%) for the IFU 2016 subgroup (p=0.577).

#### Migration

In follow-up there were 12 (7.1%) IFU 2013 patients demonstrating migration (9 cases ≥10 mm and 3 <10 mm requiring conversion). Two of the 12 were observed at 1 year and the remaining 10 between 1 and 2 years. Among the 9 IFU 2013 cases with migration >10 mm, 4 had a proximal endoleak; 3 of the 4 had aneurysm growth >5 mm.

Caudal movement between 5 and 10 mm was observed in 25 (14.9%) patients within the IFU 2013 group and 4 (8.3%) within the IFU 2016 subgroup (p=0.241). The 3 cases requiring a reintervention were described above. Between 30 days and 1 year, 10 cases of caudal movement were observed in the IFU 2013 cohort (4 in the IFU 2016 subgroup). The remaining 15 IFU 2013 cases of caudal movement were observed between 1 year and 2 years. The freedom from migration estimates (Figure 2D) were 98.3% (95% CI 95.9% to 100%) at 1 year and 89.9% (95% CI 83.0% to 96.8%) at 2 years in the IFU 2013 cohort and 100% at both time points for the IFU 2016 subgroup (p=0.150).

Patients having migration or caudal movement in combination with endoleak and/or sac growth are described in Table 7. Endoleak was found in conjunction with 5- to 10-mm caudal movement in 7 (4.2%) patients; 5 also had aneurysm growth. All cases with caudal movement in combination with endoleak and/or aneurysm growth were within IFU 2013 but not within IFU 2016.

**Table 7.** Distal Migration and Caudal Movement in Combination With Endoleak and/or Aneurysm Growth.^a^

Combinations	Distal Migration	Caudal Movement
Aneurysm growth and endoleak	2	2
Endoleak (no aneurysm growth)	1	1
Aneurysm growth (no endoleak)	3	4
Neither aneurysm growth nor endoleak	6	18
Total	12	25

a Distal migration refers to movement >10 mm or ≥5 mm with reintervention for migration; caudal movement refers to 5- to 10-mm migration.

### Aneurysm Growth

Ten (6.0%) patients demonstrated aneurysm growth in the IFU 2013 cohort, of which 2 (4.2%) were in the IFU 2016 subgroup. No aneurysm growth was seen within 30 days. Only 2 cases were observed between 30 days and 1 year (IFU 2013); the remaining 8 were between 1 and 2 years (Table 6). Freedom from aneurysm growth estimates (Figure 2E) were 97.9% (95% CI 95.5% to 100%) and 91.8% (95% CI 86.5% to 97.1%) at 1 and 2 years, respectively, in the IFU 2013 cohort and 100% and 89.3% (95% CI 77.7% to 100%), respectively, in the IFU 2016 subgroup (p=0.843).

### Survival

During the observation period, 14 (8.3%) patients died (1 at 30 days, 6 at 1 year, and 7 at 2 years) within the IFU 2013 cohort; 4 (8.3%) were in the IFU 2016 subgroup (2 at 30 days and 2 at 1 year). Of the 4 (2.4%) aneurysm-related deaths, 1 patient suffered a hemorrhagic stroke on day 16, the patient with an aortoenteric fistula at 3 months succumbed to acute renal failure and bacteremia after conversion, the patient with secondary rupture died after conversion to open repair following an unsuccessful Nellix-in-Nellix procedure, and a fourth patient died due to poor cardiac condition after open repair for a type Ia endoleak (this patient was within both IFUs). Other causes for death were cardiac illness (n=3), cancer (n=2), cerebral bleeding (n=1), bowel ischemia (n=1), dementia and pneumonia (n=1), and 2 unknown causes.

The overall survival estimates (Figure 2F) at 1 and 2 years were 95.5% (95% CI 92.2% to 98.8%) and 90.9% (95% CI 86.5% to 96.0%), respectively, in the IFU 2013 cohort and 95.5% (95% CI 89.4% to 100%) at both time points in the IFU 2016 subgroup (p=0.472). The freedom from aneurysm-related mortality estimates were 98.8% (95% CI 97.0% to 100%) and 97.6% (95% CI 94.9% to 100%) at 1 and 2 years in the 2013 cohort and 100% for both time points in the 2016 subgroup (p=0.351).

### Learning Curve and Seal Length Subanalyses

When comparing the first 15 cases within IFU 2013 in each center [n=45; median follow-up 30 months (IQR 23, 35)] to the remaining cases [n=123; follow-up 18 months (IQR 12, 24)], significantly more reinterventions were seen in the first group [6 (13.3%) vs 9 (7.3%), p=0.005], largely due to stenoses/occlusions. No significant difference between the early phase and the later phases was seen regarding the rate of any endoleak (p=0.098), migration (p=0.259), aneurysm growth (p=0.094), stenosis or occlusion (p= 0.347), or death (p=0.371).

The median infrarenal neck length prior to EVAS was 18 mm (IQR 12, 31) in the IFU 2013 group and 20 mm (IQR 15, 31) in the IFU 2016 subgroup. Seal length after EVAS, however, was 14 mm in both groups (IFU 2013 IQR 7, 25; IFU 2016 IQR 11, 24). Examining the influence of seal length on complications, the infrarenal neck length prior to EVAS was 21 mm (IQR 12, 32) in the cohort without a complication vs 16 mm (IQR 13, 23) in the group with a complication (p=0.084). The corresponding proximal seal lengths after EVAS were 14 mm (IQR 6.5, 27) when no complication was observed and 11 mm (IQR 7, 18) when a complication was present (p=0.068).

## Discussion

The present study shows that EVAS applied according to the 2013 and 2016 IFUs achieved acceptable clinical results at up to 2-year follow-up. However, the revised IFU significantly reduced the applicability of the technique. Among the 264 elective EVAS procedures analyzed in this study a mere 18% met the current anatomical characteristics of the IFU 2016. Furthermore, a remarkable number of Nellix devices were deployed outside both IFUs in our cohort. Though these were not analyzed, several reasons may have led to the use of EVAS in these patients, including ch-EVAS in patients not suitable for fenestrated EVAR, patients not fit for open repair, or patients with failed EVAR. Results in the patients outside the IFUs may well differ from the current study groups. Besides the change in IFU, several modifications in the procedure protocol were implemented during the study at different time points in the 3 centers. For these reasons, the study groups were not entirely homogeneous despite the patients being within the IFU. Moreover, because of the small number of patients within the IFU 2016 subgroup and the relatively short duration of follow-up, the results from this cohort should be interpreted with caution.

Previous studies on EVAS report reintervention rates of 0% to 12%,^8,11–17^ and a meta-analysis of reinterventions after EVAR showed reintervention rates ranging from 1.9% to 11.6%^28^ during a follow-up of up to 6 years; our reintervention data are in line with both. The 6 cases of conversion in the IFU 2013 cohort might suggest that complications after EVAS may require another approach compared to sequelae after EVAR. This may be particularly true in a center’s early experience when treatment options for complications still needed to be explored. The reintervention rate in our study was also influenced by the learning curve, with a significant reduction in reinterventions after the first 15 cases.

Apart from a learning curve, technical maturation likely contributed to better outcomes. The current technique differs considerably from the procedure that was applied when the EVAS device was just introduced into practice. The Nellix balloons are currently reinflated during the curing of the polymer or left inflated throughout the procedure. These enhancements have been introduced to avoid thromboembolic complications and enlarge the flow lumen by preventing pillowing. In addition, the need to use the entire infrarenal neck is appreciated nowadays but was not in the early phase of the observation period. This was reflected in our cohort by only a part of the available neck length being used for seal. Notably, patients with a seal-related complication had a shorter seal length, although this was not statistically significant. However, the calculated seal length in the current study could be an overestimation as it suggests an optimal circumferential wall apposition of the endobags. Further studies examining the endobag position instead of the stent position are indicated. The learning curve and improvement of best practice should be taken into account when interpreting the results.

Evaluation of endoleak, migration, and AAA growth after a Nellix procedure can be challenging. Holden et al^29^ described that a type I endoleak may be very subtle due to the device design and difficult to differentiate from calcified atheromatous plaque or contrast in the endobag. Even though duplex is a valuable tool, CTA imaging remains the gold standard, partly because migration is hard to evaluate on duplex. Additionally, noncontrast and arterial phase CT studies must be obtained to ensure optimal evaluation of the endoleak. A retrospective 3D analysis of CT was performed to ensure all endoleaks were detected; the results were discussed with the research group, which enabled scoring of the endoleaks according to a new classification that is not yet in common practice.^26^ In accordance with previous EVAS studies, type Ia was the predominant type of endoleak, while other types were extremely rare. This may be due to the fact that the endobags completely fill the aneurysm, reducing the risk of a type II endoleak, which is the most prevalent endoleak after EVAR. Proximal endoleaks were treated by embolization, proximal Nellix-in-Nellix extensions, or conversion. The durability of embolization^30^ and Nellix-in-Nellix proximal extensions^31^ remains to be shown.

The majority of migration cases were identified between 1 and 2 years, which shows that migration is a problem that occurs later in follow-up. Caudal movement of 5 to 10 mm was observed in 15% of the patients; whether this caudal movement is self-limiting or leads to progressive migration remains to be shown. However, it is known that migration >10 mm is a risk factor for other complications.^32^ England et al^33^ showed caudal migration ≥4 mm in 6 (17%) of 35 Nellix stent-grafts at 1 year in a small cohort of 18 patients, but no sequelae were mentioned. Spanos et al^34^ reported post-EVAR migration of ≥5 mm in 8.6% of cases and 6.3% when a definition of 10 mm or more was used. Within their 36-month follow-up, 22.4% of patients with migration presented with a type Ia endoleak. These observations emphasize the need for longer follow-up with 3D reconstructed CTA to enable clinicians to evaluate migration thoroughly, and follow-up should be standardized, including a noncontrast CT, to ensure endoleaks are observed if present.

The overall 8.9% mortality in this study is in accord with previous research (0.7%–9.5%^8,11–17^) despite a longer follow-up. One patient died due to multiorgan failure after undergoing a conversion to open repair for an aortoenteric fistula, which is an interesting complication of EVAS. Currently, the available evidence does not reflect a higher incidence of aortoenteric fistula after EVAS^11,15–17^ compared with EVAR. However, experience is still limited, and the incidence of fistula is too low to relate this to a statistically powerful conclusion.

It appears that the refined 2016 IFU does not clearly lead to a better outcome after the EVAS procedure compared to the 2013 IFU. Although not statistically significant, the prevalence of complications was lower in the IFU 2016 group. However, this may be related to the small sample size in the IFU 2016 cohort. There were no significant differences in the incidence of endoleak and aneurysm growth between both IFUs, and there were no migrations observed in the 2016 cohort. Nevertheless, the data may not reflect current practice since a second-generation device was introduced in April 2016. One of the major changes is that the endobag is now attached not only proximally but also distally. In the first-generation device the distal part of the endobag could migrate proximally during deployment, and insufficient distal seal is considered one of the root causes of aneurysm growth.^35^

### Limitations

This was a retrospective study with data collected as available, and therefore some data were missing. The small sample size of the IFU 2016 group might be a cause for bias, and the median follow-up duration in this study was 23 months. All CT scans were assessed by a single researcher with an extensive experience in post-EVAS imaging using core laboratory protocols that have been shown to have a very good interobserver variability.^36^ In addition, all complications were studied by 3 vascular surgeons to improve the quality of data. In some cases, the latest available imaging modality was duplex ultrasound, so some cases of migration might have been missed. The study comprised all patients, including the first EVAS procedures, in the 3 sites so a learning curve effect is likely. Also, this research includes solely the outcomes of the Nellix device when used within the IFU. Likely, this study presents more positive results as compared to the entire cohort, but this goes beyond the scope of this study.

## Conclusion

The present study showed that EVAS applied within the IFU has acceptable 2-year results, but longer follow-up and a larger sample size are clearly indicated. The refined IFU significantly reduced the applicability of the technique.
